# Weight loss in combat sports: physiological, psychological and performance effects

**DOI:** 10.1186/1550-2783-9-52

**Published:** 2012-12-13

**Authors:** Emerson Franchini, Ciro José Brito, Guilherme Giannini Artioli

**Affiliations:** 1 Martial Arts and Combat Sports Research Group, School of Physical Education and Sport, University of São Paulo, Sao Paulo, Brazil; 2 Center for Research in Sport Performance and Health (NEDES), Federal University of Sergipe, Sergipe, Brazil; 3 Laboratory of Applied Nutrition and Metabolism, School of Physical Education and Sport, University of São Paulo, Sao Paulo, Brazil

**Keywords:** Martial arts, Rapid weight loss, Athletic performance, Diuretics, Energy restriction, Weight cycling

## Abstract

**Background:**

The present article briefly reviews the weight loss processes in combat sports. We aimed to discuss the most relevant aspects of rapid weight loss (RWL) in combat sports.

**Methods:**

This review was performed in the databases MedLine, Lilacs, PubMed and SciELO, and organized into sub-topics: (1) prevalence, magnitude and procedures, (2) psychological, physiological and performance effects, (3) possible strategies to avoid decreased performance (4) organizational strategies to avoid such practices.

**Results:**

There was a high prevalence (50%) of RWL, regardless the specific combat discipline. Methods used are harmful to performance and health, such as laxatives, diuretics, use of plastic or rubber suits, and sauna. RWL affects physical and cognitive capacities, and may increase the risk of death.

**Conclusion:**

Recommendations during different training phases, educational and organizational approaches are presented to deal with or to avoid RWL.

## Background

Combat sports represent ~25% of the Olympic medals. Certain sports (e.g., boxing and mixed martial arts) are watched by millions of spectators [1,2]. In almost all combat sports, athletes are classified according to their body mass so the matches are more equitable in terms of body size, strength and agility [3,4]. However, many athletes acutely reduce body mass in an attempt to get an advantage by competing against lighter, smaller and weaker opponents [4,5]. Despite the well documented adverse effects of rapid weight loss (RWL) on health status, the prevalence of aggressive and harmful procedures for rapid weight reduction is very high in most combat sports, such as wrestling [6], judo [5,7-10], jujitsu [10], karate [10], taekwondo [10-12] and boxing [13]. Although there is no controversy on literature regarding the negative impact of RWL on physiological and health-related parameters [14], the effects on competitive performance are somewhat equivocal, as many factors (e.g., time of weight reduction, recovery time after weigh-in and type of diet) may affect responses to weight loss.

In this narrative review (performed in the databases MedLine, Lilacs, PubMed and SciELO), we discuss the most relevant aspects of RWL in combat sports, namely *(1)* the prevalence, magnitude and procedures used; *(2)* the effects of weight loss on psychological, physiological and performance parameters; *(3)* strategies to avoid performance decrements and *(4)* organizational strategies to avoid harmful practices among athletes.

### Rapid weight loss: prevalence, magnitude and procedures

Several studies have reported high prevalence of RWL (60–90% of competitors) among high school, collegiate and international style wrestling [6,15,16]. In judo, a similar trend was found, as ~90% of athletes (heavyweights excluded) reported that they have already reduced body weight rapidly before a competition and a somewhat lower percentage reduce body weight before competing on a regular basis [5]. Brito et al. [10] reported a slightly lower percentage of judo athletes regularly reducing weight (62.8%), which was similar to athletes from jujitsu (56.8%), karate (70.8%), and taekwondo (63.3%). The percentages found in all these sports are comparable to the range previously reported in wrestlers.

Gender is not a factor affecting the prevalence of RWL, although competing at a higher levels was related with more aggressive weight management strategies [5]. However, a recent study [10] showed that competitive level is not associated with weight management behaviors in jujitsu, judo, karate and taekwondo athletes. Of concern, ~60% of judo athletes started reducing weight rapidly before competitions at very early ages (i.e.,12–15 years) [5], which was also observed in Iranian wrestlers (15.5 ± 2.4 years) [17]. Brito et al. [10] also reported that RWL begins during adolescence in karate and taekwondo athletes (13.6 ± 1.4 and 14.2 ± 2.1 years, respectively). On the other hand, jujitsu athletes started reducing weight somewhat later (21.1 ± 5.2 years). Evidence shows that weight cycling during adolescence can be a major issue, as it might negatively impact growth and development [18]. Importantly, it has been suggested that athletes beginning to cut weight at early ages are at higher risk of weight loss-related problems [5].

It is worthy to note that the range of body weights of the various weight classes in sports recently included in the Olympics (e.g., female: boxing, wrestling and taekwondo) are considerably broader than the range of those sports with longer tradition in the Olympic Games (e.g., boxing and judo). While the range of the more recent Olympic sports varies around 15%, the difference of the upper limit between two consecutive categories varies around 5–10% in boxing and judo. Thus, an athlete with a body mass at the midpoint of two weight classes in judo and boxing would be more tempted to reduce his/her body mass to a lower class, whilst an athlete in the same condition, but competing in taekwondo, would be less prone to move to lighter class, as the reduction would be more dramatic. However, no study was conducted so far in order to compare weight management behaviors between those combat sports.

With regard to the magnitude of weight loss, although most athletes reduce body weight in a range of 2–5%, a considerably high percentage (i.e.,~40%) reduces 5–10% of their body weight [5,6]. Furthermore, most athletes reported that their greatest body weight reduction was of 5–10%; however, many athletes reported reductions of more than 10% of body weight [5,6,10]. Such reductions are frequently undertaken in a few days before competitions. In most cases, athletes reduce weight in the week preceding the weigh-in [5,6,15]. The Table 1 summarizes the main findings of the studies on the prevalence and magnitude of weight loss in combat sports.

**Table 1:** Weight loss prevalence and magnitude in combat sports’ athletes

**Sample**	**Prevalence**	**Magnitude**	**Authors**
Brazilian judo (n = 145)	Males: 62.8%	Males^a^: 5.6 ± 2.2 kg	Brito et al.[10]
		8.5 ± 4.2%	
Brazilian jujitsu (n = 155)	Males: 56.8%	Males^a^: 2.9 ± 1.5 kg	
		4.1 ± 2.0%	
Brazilian karate (n = 130)	Males: 70.8%	Males^a^: 2.5 ± 1.1 kg	
		3.6 ± 2.2%	
Brazilian taekwondo (n = 150)	Males: 63.3%	Males^a^: 3.2 ± 1.2 kg	
		4.3 ± 3.2%	
Iranian wrestling (n = 436)	62%	3.3 ± 1.8 kg (5.0 ± 2.6%)	Kordi et al.[17]
Brazilian judo (n = 822)	86% (all categories)	Most of the athletes reduced between 2–5%	Artioli et al.[5]
	89% (heavyweights excluded)		
Brazilian judo (n = 105 males and 20 females)	Males: 77.1%	Males: 4.5 ± 3.5 kg	Fabrini et al.[19]
	Females: 55.0%	Females: 1.7 ± 0.8 kg	
USA judo (n = NR)	70–80%	NR	Horswill[20]
Brazilian Olympic Boxing Team	100%	5.8 kg	Perón et al.[13]
Canadian taekwondo (n = 28)	53%	NR	Kazemi et al.[11]
USA high school wrestling (n = 2352)	62%	2.9 ± 1.3 kg	Kinigham and Gorenflo[21]
		4.3 ± 2.3%	
USA college wrestling (n = 63)	89%	5 kg	Steen and Brownell[6]
USA high school wrestling (n = 368)	70%	2.3 kg	
USA high school wrestling (n = 747)	NR	3.1 ± 2.4 kg	Tipton and Tcheng[22]

NR = not reported; a = weight loss for the week before competition.

To achieve such a rapid weight reduction, athletes use a variety of methods [4,5,7,10,15], such as: reduced liquid ingestion; use of saunas, blouses and plastic suits; reduced energy intake; fasting one day prior to the weigh-in; reduced carbohydrate and fat intake. Other more aggressive methods are also used, such as [23] vomiting, diet pills, laxatives and diuretics. It is important to emphasize that diuretics are prohibited by the World Antidoping Agency [24] and are responsible for the majority of doping cases in combat sports [25].

### Psychological effects of rapid weight loss

Several investigations have reported that athletes undergoing RWL presented decreased short-term memory, vigor, concentration and self-esteem as well as increased confusion, rage, fatigue, depression and isolation [6,26-29], all of which may hamper competitive performance. For example, decreased short-term memory can impact the ability of an athlete to follow his/her coach’s instructions before a match. Likewise, the lack of concentration and focus can affect the ability of the athlete to deal with distractions during high-level competitions, resulting in poor performance. A low self-esteem may result in difficult to consider the possibility of winning a match, especially against high-level opponents. Confusion can negatively affect the capacity of making decisions during the match and rage may result in lack of control and, despite the importance of aggressiveness for combat sports, excessive rage may increase the possibility of illegal actions. Depression and isolation can result in difficulty in coping with rigorous training sessions.

In addition to these problems, a high percentage of wrestlers are quite concerned about their body mass and food intake. Consequently, they resort to frequent dieting or caloric restriction. Of great concern is the fact that 10–20% of them feel unable to control themselves while eating, which is a classic symptom of an eating disorder. This number increases to 30–40% after the competition [6]. The constant attention directed to body mass control increases the probability of eating disorders such as binge eating, anorexia and bulimia, with higher risk among female athletes [23,30]. In fact, wrestlers present preoccupation about their body mass and are not satisfied with their body, despite the very low body fat percentage they usually present. This behavior appears to be more marked in athletes competing at higher levels [31]. Not surprisingly, the prevalence of overweight and obesity are higher in former combat athletes in comparison with former athletes who were not weight cyclers during their competitive career [32].

### Rapid weight loss and competitive success

A few studies investigated the association between RWL and competitive success in real tournaments [16,33,34]. Although competitive success is multifactorial and too complex to be determined by one variable, the associations provided by these investigations are insightful and help discern the impact of RWL on competitive performance.

In a regional-level wrestling competition, it was observed that athletes who lost a higher amount of weight achieved better classification than the athletes who lost less weight [34]. When all weight categories were grouped, a higher percentage of medalists (58%) had not followed the minimum wrestling weight recommendations compared to those who had followed such recommendations (33%). Thus, athletes who had practiced more aggressive weight cutting procedures presented better competitive results as compared to those who were more conscious with their health.

Studies performed in national level competitions have produced conflicting data. In a study by Horswill et al. [33], the amount of body mass recovered after the weigh-in and the success in a wrestling competition were recorded. No differences in absolute weight gain were observed between winners and defeated athletes (winners = 3.5 ± 1.2 kg; defeated = 3.5 ± 1.5 kg). The authors also observed no influence of relative weight gain (winners = 5.3 ± 2.0%; defeated = 5.3 ± 2.4%) and weight difference between the athlete and his opponent (winners = 0.1 ± 2.0 kg; defeated = −0.1 ± 2.0 kg) on success [33]. Assuming that the body mass recovered after weigh-in is associated with body mass reduced before the weigh-in, the authors concluded that the amount of weight lost and, consequently, the amount of weight regained after the weigh-in has no effect on competitive success. In contrast, Alderman et al. [16] reported that winners reduced a higher amount of body mass (mean reduction = 3.78 kg; range = 2.95–4.77 kg) compared to defeated athletes (mean reduction = 3.05 kg; range = 1.91–3.95 kg).

Some authors [8] argue that a successful career is probably built in a single weight class. By changing to a different weight class, a given athlete may have to pass through a complex adaptive process because he/she would face completely different opponents with different fighting styles. Thus, it seems intuitive that an athlete wants to compete in the same weight class for as long as he/she is able to make that weight. Despite the paucity of evidence that indicates an association between rapid weight loss and competitive success [5,14], it must be noted that it is possible to achieve success in combat sports while competing in multiple weight classes. Some prime examples are the successful athletes who moved to heavier weight classes and still performed at the highest level (e.g., Ilias Iliadis, João Derly, Leandro Guilheiro, Keiji Suzuki, Tsagaanbaatar Khashbaatar, Sun Hui Kye, Oscar de la Hoya, Evander Holyfield, Manny Pacquiao). While studies are scarce and inconclusive, the impact of RWL on competitive success remains elusive, especially when considered the great number of variables defining wins and losses.

### Physiological effects of rapid weight loss

Despite conflicting evidence, most studies indicate that weight loss decreases both aerobic and anaerobic performance. While aerobic performance impairments have been attributed to dehydration, decreased plasma volume, increased heart rate, hydroelectrolytic disturbances, impaired thermoregulation and muscle glycogen depletion [30], decreased anaerobic performance is mainly related to reduced buffering capacity, glycogen depletion and hydroelectrolytic disturbances [30,35]. Maximal strength seems to not be acutely affected by RWL [36-38], although chronic weight cycling has a negative impact on strength gain during a season [39].

It is important to highlight that the decrements on anaerobic performance are generally observed when athletes have no opportunity to refeed and rehydrate after weigh-in [27,38,40,41]. However, in the most combat sports competitions, weigh-ins are followed by a period of time during which athletes may have the chance to recover from the weight loss. Although this period may vary from a few hours to more than one day, it is very likely that within 3–4 hours, athletes are able to recover their anaerobic performance to pre-weight loss values [9]. Therefore, when followed by a relatively short recovery period, RWL will probably have minimal or no impact on anaerobic performance. Although this seems to be true for athletes who are experienced weight cyclers, athletes with no experience in reducing weight might be negatively affected by weight loss [42,43]. It suggests that weight cycling may lead athletes to develop physiological adaptations that help them to preserve performance after weight loss. However, to date there is no direct evidence supporting these hypothesis and further studies are needed to confirm or refute them.

Some epidemiological studies have associated RWL with increase risk for injuries [44]. Oöpik et al. [45] observed that the 5% reduction in body mass affected metabolism and muscle contraction pattern, thereby increasing exposure to injury. One study revealed that athletes who had reduced more than 5% of their body mass presented a higher probability of injury during competition [46].

### Extreme cases

Due to the possible adverse effects of RWL, there are rare cases of death related to this practice. In 1996, just three months before Atlanta Olympic Games, Chung Se-hoon (22 years, 74 kg), considered the probable gold medal winner in the 65 kg weight category in judo, was found dead in a sauna. The c*ausa mortis* was a heart attack. One year later, three collegiate wrestlers died due to hyperthermia and dehydration associated with intentional RWL [47]. During the Sydney Olympics, Debbie Allan from Great Britain was disqualified during the weigh-in because the scale used by her was not calibrated due to an alleged scale sabotage [48]. The problem seems also to affect children. In 2005, Sansone and Sawyer [49] reported weight loss pressure on a 5-year-old wrestler, whose father was asking him to lose 10% of his body mass to take part in a wrestling tournament. Those extreme cases, together with the very high prevalence of RWL achieved by aggressive methods, illustrate quite clearly that the scenario is disturbing, the problem may be more serious than many people involved with the sport may think and that more attention to this problem should indeed be given.

### Strategies to avoid decreased performance after rapid weight loss

No athlete should be encouraged to cut weight quickly in order to compete in a lighter weight class. Although performance may not be affected, an athlete’s health is always at risk. If an athlete needs to adjust his/her body weight, there are strategies that one can follow to help minimize the potential adverse effects: [14,20,50-52]:

1) Gradual weight loss (i.e.,<1 kg.week^−1^), rather than RWL, must be the preferential method for adjusting weight.

2) Athletes should aim to maximize body fat loss and minimize muscle wasting and dehydration when adjusting weight.

3) An athlete who needs to reduce more than 5% of body weight should consider not losing weight.

4) An athlete who needs cut weight so that his/her body fat would lower than 5% for men and 12% for women should consider not losing weight.

5) During the weight loss period, strength training and BCAA supplementation may help preserve muscle mass.

6) Athletes should not undergo low-carbohydrate diets in order to make weight as they seem to be more detrimental to physical performance [41].

7) If an athlete will have less than 3 hours to recovery after the weigh-in, RWL, dehydration and restricted carbohydrate ingestion should be avoided.

8) During the recovery period after weigh-in, athletes are encouraged to consume high amounts of carbohydrates, fluids and electrolytes. Creatine supplementation may also be of use if the athlete will recover for a long period after weighing-in.

### Management strategies to avoid rapid weight loss practices

Control strategies to avoid RWL practices can be divided in two areas: (1) coach and athlete educational programs; (2) management procedures to control or discourage RWL.

### Coach and athlete educational programs

Considering that most athletes follow their coaches’ recommendations to execute RWL [5,8,17], the best strategy is to make both coaches and athletes fully aware of the risks involved with RWL and the recommended procedures to gradually reduce body mass [17]. According to Burke and Cox [3], athletes and coaches should receive information about: caloric balance; how to prepare each food portion; how to avoid increase weight (especially fat) after the competition; how to prepare food using low fat ingredients; how to prepare snacks with low caloric content using fruits and vegetables; how to avoid combating stress through excessive food intake; how to avoid gastronomic novelties during high-level competitions abroad or when inside the Olympic village; the importance of avoiding fast-food restaurants while travelling; how to increase satiety using low glycemic index foods; how to avoid excessive food and alcohol intake during celebrations; how to keep a diet diary and how to identify the main difficulties to maintain adequate nutrition. Additionally, the recommendations done by Horswill [20] concerning body mass control during the season are important sources of information. This author suggests specific goals for each periodization phase. Pre-season: determine athlete’s optimal weight category; estimate body composition to determine the minimum body mass the athlete can have to compete safely; initiate the weight category change if needed; adjust technique and tactics for the new weight category; aerobic conditioning and strength training to reduce body fat and maintain muscle mass; reduce energy and fat intake to decrease body fat percentage; Season: keep body mass near the upper weight limit; increase caloric intake to deal with training and competition demands; maintain strength training; adequate micro and macronutrients intake; Off season: avoid increase in body fat; begin strength training; maintain aerobic conditioning; avoid high-fat diets.

### Management procedures to control or discourage rapid weight loss

Management procedures have been used in wrestling [53] and proposed for judo [8] to avoid weight loss among athletes. The following recommendations were first drafted in 1976 [54] and reinforced in 1996 by the American College of Sports Medicine [14]. They are currently in use in most scholastic wrestling competitions in United States as a part of a program aiming at controlling the weight management issue among wrestlers. This program has been shown effective in attenuating the aggressive patterns of rapid weight loss and discouraging athletes from losing weight irresponsibly [20]. Therefore, these recommendations should be implemented by other combat sports organizations in order to avoid widespread weight loss among combat athletes [8]:

– matches should begin in less than 1 h after weight in;

– each athlete is allowed to weigh-in only one time;

– RWL methods and artificial rehydration methods are prohibited on competition days;

– athletes must pass the hydration test to get the weigh-in validated;

– an individual minimum competitive weight is determined at the beginning of each season;

– no athletes are allowed to compete in a weight class that would require weight loss greater than 1.5% of body mass per week.

## Abbreviations

RWL: Rapid weight loss.

## Competing interests

The authors declare they have no competing interests regarding this manuscript.

## Authors’ contributions

All authors have written the first draft of the manuscript, revised it and approved its final version.
